# Effects of an enhanced iron dense foods offering in the daily meals served in geriatric institutions on measures of iron deficiency anemia

**DOI:** 10.1186/s12877-018-0800-9

**Published:** 2018-05-25

**Authors:** Baerbel Sturtzel, Ibrahim Elmadfa, Brigitte Hermann, Walter Schippinger, Gerald Ohrenberger

**Affiliations:** 1 Department of Nutritional Sciences, University of Vienna, Althanstraße 14, 1090 Vienna, Austria; 2 Haus der Barmherzigkeit, Long term care hospital, Seeböckgasse 30a, 1060 Vienna, Austria; 3 Albert Schweitzer Hospital, Long term care hospital, Albert-Schweitzer-Gasse 36, 8020 Graz, Austria

**Keywords:** Iron deficiency anemia, Geriatric patients, Oral iron intake, Long-term-care

## Abstract

**Background:**

Iron deficiency is one of the most common causes of anemia in geriatric patients. Although the oral iron intake is often inadequate, the potential of iron dense foods in the daily meals of geriatric institutions is rarely considered. To test during a 1- year span whether an improved frequency of iron dense foods in the daily meals has an impact on the oral iron intake, the hemoglobin concentration and anemia prevalence of institutionalized geriatric patients. A parallel, open, pre-and post-oral nutrition intervention study. Two geriatric hospitals participated as intervention centers and one as comparison center.

**Methods:**

In the two intervention centers, a menu plan adapted with iron dense foods was applied. In the comparison center the regular meals provisions was continued. At months 1, 6 and 12 of the intervention time the routine blood-parameter hemoglobin was taken from the geriatric hospital’s medical report. Component analysis assessed the nutrient density of the offered meals. 2-day-weighing records realized at month 1 and 6 of intervention-time assessed the iron intake. Ninety-nine geriatric patients in the intervention centers and 37 in the comparison center. All of them had multiple chronic diseases and an average age of 84 years. With the non-parametric Friedmann-Test for repeated measurements, we establish differences within the groups. With the Mann-Whitney-U-Test, we establish differences between the groups. For dichotomous variables, the chi-square-test was used. A *p*-value of< 0.05 was considered statistically significant for all analyses.

**Results:**

In the intervention centers the iron intake (*p* < 0.001) and the hemoglobin concentration (*p* = 0.002) improved significantly (p < 0.001). As in the comparison center the frequency of meat and sausage offerings was twice as much as recommended also the hemoglobin concentration improved (*p* = 0.001).

**Conclusion:**

Geriatric patients with anemia or low hemoglobin level benefit optimally from a diet rich in iron dense foods. Enhanced access to such can indeed correct iron deficiency anemia.

**Trial registration:**

The ethics committee of the Municipality of Vienna (EK-13-043-0513) approved the study.

## Background

Anemia is common in the elderly and is independently associated with increased morbidity and mortality [1]. The WHO defined anemia as a hemoglobin level lower than 13 g/dl in men and less than 12 g/dl in women [2]. The highest prevalence of anemia was found in geriatric patients and nursing home residents [3]. Iron deficiency is one of the most common causes of anemia in the elderly [4].

Iron deficiency anemia evolves when the body-stored iron is low [2]. Iron deficits can be remedied with iron supplements or iron dense foods [2]. However, for rectifying iron deficits, iron dense foods should be preferred. Even more, as it is well established that low oral iron intake and low bioavailability of dietary iron are the prime culprits for the development of iron deficiency anemia [5]. In addition, scientific reference bodies like the Dietary Guidelines Advisory Committee of the United States emphasize the importance of healthy, food-based dietary patterns [6].

Iron dense foods can be of plant or animal origin [7]. The bioavailability of iron from plant based foods is broadly discussed since its digestibility is affected by several dietary components like phytic acid or polyphenols, but primarily as iron here is not heme bound [8, 9]. Good sources for plant-based iron are for example lentils, pine seeds, nuts or linseed and oat bran. By contrast, the bioavailability of heme iron of animal-based foods is much better [7]. It is absorbed more readily [9] via integral membrane transporter proteins in the intestinal cells [10]. Heme iron rich foods are meats or meat products like sausages and giblets such as liver.

The reported iron deficit in geriatric patients results from the fact that catering services offers iron dense foods less frequently and if they do, the offered amount is generally not adapted to the nutritional needs of the patients [11]. It is also well documented that a food consumption lower than the recommended daily intake is common in geriatric institutions and therefore the quality of the daily meals should be adapted to the nutritional needs [11, 12]. Particularly as a low food-consumption may cause an imbalance between nutrient intake and dietary requirements [12, 13]. Therefore, beside the failure to absorb iron or an interaction of iron with drugs, an imbalance between iron intake and need may lead to iron deficiencies [14], to a decreased hemoglobin synthesis and finally to iron deficiency anemia [14].

While poor oral intake has so far been a potentially remediable risk factor for poor nutrition in geriatric institutions [11], there is no information on how to remedy low iron intake and how to raise low hemoglobin concentrations with iron dense food items [15, 16] offered by the catering services.

The causality between a low iron intake, low hemoglobin concentration and the onset of iron deficiency anemia in geriatric institutions is all too obvious. Only follow-up or intervention-studies will allow an assessment of this interlink [17].

For bridging this gap, we designed the present study. Particularly as it was shown [13] that the oral iron intake of geriatric patients was inadequate due to the lack of iron dense foods. Therefore, we concluded that we should modify the menu plan and integrate iron dense foods in the daily meals. We supposed that these provisions should lead to an improved oral iron intake and consequently to higher hemoglobin concentration and lower anemia prevalence among the elderly.

Objective: To test whether during a 1- year span an improved frequency of iron dense foods in the daily meals will have an impact on the oral iron intake, the hemoglobin concentration and anemia prevalence of institutionalized geriatric patients.

## Methods

### Design

**Fig. 1:**
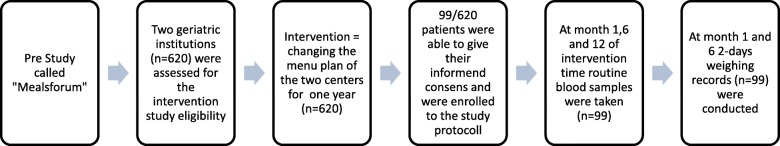
Flow chart of the intervention

A parallel, open, single group, pre-and post-oral nutrition intervention study. For the duration of 12 months two geriatric hospitals participated as intervention-centers (see Fig. 1). For the comparison of the menu plan habits, an additional center provide their information.

### Preparation of the line of action

Based on literature [18–20] we assumed that geriatric patients would not always like menus with iron dense foods and that it would be necessary to spread the iron dense foods over the entire menu plan period of 8 weeks. In addition, in order to avoid an over-offering of one recipe, we switched between various recipes of iron dense foods. The study lasted 12 month.

Three months before the start of the intervention, we convened so-called “Meal-Forum” in order to develop the intervention strategy and implementation protocol. Recipes of animal-based iron dense foods such as liver, black pudding and plant-based iron dense foods like oat-bran or legumes (for example dried peas, edible beans, lentils or chickpeas) were drawn up. Moreover, it was also established that whenever a meal with iron dense foods would be offered a parallel-menu without such should be offered and that at any time patients would have the option of independently choosing a meal of their liking.

### Iron density of the recipes

It was shown that the iron intake of geriatric patients through the daily meals was on average 6.9 mg/d [19, 20]. According to the guidelines of the nutrition societies (e.g. D-A-CH Reference Values (21)) this is not enough to sustain a satisfactory iron status which is expected to be met at a recommended daily intake of 10 mg iron [21]. To rectify this imbalance a supplement of 3.1 mg iron/day/person from iron dense foods was needed. Therefore, it was thus calculated that, once/week a soup with liver dumplings (pork liver circa 7 mg iron), a dinner with chicken liver (chicken liver circa 11 mg iron) and a supper with black pudding (pork sausage, circa 6 mg iron) should be integrated into the daily meals. We reckoned that through this extra-supplement of iron, an additional intake of 24 mg iron/week or 3.5 mg/day (24:7) should be achieved. To ensure an optimal iron offering we also provided plant based iron in form of oat-bran and lentils. Thus, a cake with oat-bran (circa 1.5 mg iron/day) and a lentil-spread (circa 0.6 mg iron/day) was offered twice a week. In total, we calculated, that with this approach an estimated daily optimization of circa 4, 9 mg iron/person should be achieved. This calculated additional intake was well over and above covering the lacking 3.1 mg iron/day/person, but still low enough to avoid excessive iron intake [21]. Generally, in both intervention centers the menu plan was changed for all patients. During the course of one week the recommended soup, dinner and supper with iron dense foods were offered. The iron density was calculated from the total daily iron intake (mg) and the total daily energy intake (MJ).

### Assessing the oral iron-intake

Prior to the start and after six months of intervention 2-day-weighing records were established (see Table 1). Trained staff, who weighed the plates with the meals before and after the participants’ food intakes during the course of the day, compiled them. The calculation of the iron intake was executed by means of nut.s® nutritional software based on the German Food database BLS 3.01, whereby we used the mean (±SD) of the 2-day-weighing records.

**Table 1:** Mean (±) of iron, energy and protein intake as well as iron density of the participants (*n* = 99) at the beginning (month 1) and middle (month 6) of the intervention time

	Month 1	Month 6	p-value (^a^)	Reference
Iron (mg/day)	8.94 (±2.5)	10.34 (±4.2)	< 0.001*	10
Energy (MJ/day)	5.95 (±1.4)	6.26 (±1.4)	< 0.05*	–
Protein (g/kgBW/d	0.75 (±0.29)	0.84 (±0.28)	< 0.005*	0.8
Iron Den. (mg Fe/MJ)	1.50	1.65		

(^a^) Wilcoxon Rang Test; Significance* = p < 0.05

### Assessing the hemoglobin concentration and anemia-prevalence

At the beginning (month 1), middle (month 6) and the end (month 12) of the intervention time the routine blood-parameter hemoglobin was taken from each geriatric hospital’s medical report. The hemoglobin concentration was determined at the geriatric hospital’s standard laboratory. The anemia prevalence was calculated according to the WHO definition [2]. Additionally, in order to assess the iron levels the routine blood-parameters ferritin, transferrin and iron in blood were also taken from the medical report.

### Choice of intervention and comparison centers

Each geriatric hospital has its own work procedures and guidelines, which may influence a study’s results. To be able to exclude the factor “work-related impact”, the intervention was carried out in two geriatric facilities. Likewise, to exclude the factor “center-related impact”, a third hospital served as comparison.

### Qualitative assessment of the iron intake for evaluating the results of the comparison center

According to the principles for estimating the nutritional quality of the offered meals [22] a menu plan component analysis was conducted. We compared the frequency of the food groups used in the kitchens of the intervention-centers with those of the comparison center (according the recommendations of the German nutrition society [23]). We compared the frequency of the offered food groups during one 8-week menu plan period with the recommendations [23] (Table 2).

**Table 2:** Mean (±SD) of hemoglobin concentration (g/dl) from the participants of the intervention and comparison at month 1, 6 and 12 of intervention time such as subdivided in the two subgroups without and with anemia

	Intervention Centers (*n* = 99)		Comparison Center (*n* = 37)		
Hemoglobin (g/dl)	MW (±SD)	*p*-value (^a^)	MW (±SD)	*p*-value (^a^)	*p*-value (^b^)
	All (n = 99)		All (n = 37)		
Month 1	12.78 (1.36)		12.74 (1.65)		0.934
Month 6	12.79 (1.23)		13.05 (1.33)		0.481
Month 12	13.00 (1.24)	0.352	13.05 (1.32)	0.058	0.807
	Intervention without Anemia (*n* = 70)		Comparison without Anemia (*n* = 22)		
Month 1	13.40 (1.04)		13.80 (1.09)		0.110
Month 6	13.20 (1.01)		13.65 (1.22)		0.110
Month 12	13.32 (1.14)	0.246	13.60 (1.17)	0.888	0.230
	Intervention with Anemia (*n* = 29)		Comparison with Anemia (*n* = 15)		
Month 1	11.28 (0.77)		11.20 (0.95)		0.941
Month 6	11.80 (1.16)		12.10 (0.89)		0.377
Month 12	12.24 (1.14)	0.002*	12.25 (1.13)	0.001*	0.931

(^a^) Differences within groups were tested with Friedmann Rang Analysis; (^b^) Differences between groups were tested with Mann-Whitney-U-Test; groups = centers; Significance * = *p* < 0.05

Correlation between iron intake and hemoglobin: *r* = 0.172; *p*-value: < 0.01

### Patients and ethics

All patients suffered from multiple chronic diseases and required assistance to perform their daily life activities. The ethics committee of the Municipality of Vienna (EK-13-043-0513) approved the study and their ethical guidelines were followed. The patients or their legally authorized representatives were asked for giving their written informed consent. Whenever it was possible to get a full approval to participation, we included the data into the evaluation (see Fig. 1). Since many individuals in geriatric populations suffer from dementia, we decided that also patients with early dementia (MMSE> 11) [24] should be asked to participate, mainly those who had an independent motivation and who were not fed by the nurses. We were able to get informed consent from 99 patients in the intervention centers and 37 in the comparison center. From all study participants we had the data from months 1, 6 and 12 of the intervention time. The 99 participants had a mean (±SD) age of 84.19 (±7.9) years. 86% (*n* = 85) of them were women and 14% (*n* = 14) men. The mean (±SD) body mass index of the women was 26.9 (±5.85) and that of the men 25.7 (±3.51).

### Statistical analysis

Data were analyzed using SPSS (Windows version 23.0, IBM-SPSS, Inc., Chicago, IL). As the sample size was small, we chose non-parametric tests for the statistical analysis. We showed means and standard deviations or counts and percentages. For continuous variables the non-parametric Friedman-Test and Wilcoxon-rang-test for repeated measurements was used to establish the differences within the groups. For dichotomous variables, the chi-square-test was used. A linear regression was conducted. A *p*-value of< 0.05 was considered statistically significant for all analyses.

## Results

The iron intake improved significantly (*p* < 0.001) from 8.94 (±2.5) to 10.34 (±4.2) mg/d. The iron density improved from 1.50 (mg Fe/MJ) at month 1 to 1.65 (mg Fe/MJ) at month 6 (Table 1). A weak but significant (*p* < 0.01) correlation between iron intake and hemoglobin concentration (g/dl) was found (Table 2). In anemic patients the hemoglobin concentration improved significantly (*p* = 0.002) (Table 2). Similarly, the anemia prevalence decreased by about 10%. The anemia prevalence was 29.3% at month 1 and 19.2% at month 12 (Table 3). The ferritin concentration (ng/ml) increased significantly in patients with no-anemia and the transferrin concentration (mg/dl) in patients with anemia (Table 4). The coefficient beta of the linear regression model showed that ferritin and transferrin had a negative effect on hemoglobin concentration in patients with anemia (Table 5). The menu-plan-component analysis showed that in the intervention-centers the offered food groups did correspond to the pattern recommended by the German nutrition society. In the additionally consulted comparison-center, the frequency of offering meat and sausages (both rich in heme-iron) was twice as much as recommended (Table 6). Between the intervention and comparison-centers were no differences in the mean hemoglobin concentration.

**Table 3:** Prevalence of anemia at the beginning (month 1) and at the end (month 12) of intervention time

	Intervention Centers (n = 99)		
	no anemia	anemia	p-value (^a^)
Month 1	70 (70.7%)	29 (29.3%)	
Month 12	80 (80.8%)	19 (19.2%)	0.002*

(^a^) binominal and chi-quadrat-tested; Significance* = *p* < 0.05

**Table 4:** Mean (±SD) of Transferrin (mg/dl), Ferritin (ng/ml) and Iron in Blood (μg/dl) concentration of all participants such as participants without and with anemia at month 1, 6 and 12 of intervention time

	All patients (*n* = 99)	*p*-val. (^a^)	no anemia (*n* = 70)	*p*-val.(^a^)	Anemia (*n* = 29)	*p*-val.(^a^)
Transferrin (mg/dl)						
Month 1	218.54 (±47.67)		222.91 (±41.32)		207.78 (±60.10)	
Month 6	224.09 (±44.97)		226.82 (±41.77)		217.35 (±52.24)	
Month 12	230.50 (±48.01)	0.009*	229.25 (±41.13)	0.138	233.53 (±60.78)	0.006*
Ferritin (ng/ml)						
Month 1	149.61 (±134.61)		150.28 (±133.5)		147.88 (±139.1)	
Month 6	144.79 (±125.85)		142.82 (±126.4)		149.64 (±126.6)	
Month 12	158.32 (±141.32)	0.230	164.38 (±144.4)	0.047*	143.60 (±134.7)	0.712
Iron in Blood (μg/dl)						
Month 1	70.55 (±26.65)		76.37 (±27.29)		56.51 (±19.03)	
Month 6	69.80 (±26.38)		74.72 (±26.12)		57.93 (±23.39)	
Month 12	68.00 (±23.86)	0.213	70.13 (±24.13)	0.117	62.93 (±22.82)	0.867

(^a^) Friedmann Rang Analysis; Significance* = *p* < 0.05

**Table 5:** Linear Regression model to predict hemoglobin concentration according the iron metabolism parameter iron in blood (μg/dl), transferrin (mg/dl), ferritin (ng/ml) in all participants (*n* = 99) such as the participants without (*n* = 70) and with (*n* = 29) anemia

All patients	Coefficient beta	95% CI	*p*-value
Iron in blood	0.358	0.009–0.028	0.000*
Transferrin	0.180	−0.001 – 0.011	0.119
Ferritin	0.191	0.000–0.004	0.099
Without anemia			
Iron in blood	0.151	−0-003 – 0.014	0.201
Transferrin	0.243	−0.001 – 0.013	0.092
Ferritin	0.314	0.000–0.004	0.032*
With anemia			
Iron in blood	0.085	−0.013 – 0.020	0.673
Transferrin	−0.448	−0.011 – 0.001	0.092
Ferritin	−0.489	−0.006 – 0.000	0.048*

Significance* = *p* < 0.05; Coefficient beta = the negative sign indicated a negative effect of the independent variable on hemoglobin concentration

**Table 6:** Analysis of the menu components of a one-week menu plan in the intervention- and comparison centers

	Intervention	Comparison	Referenz (D-A-CH)^a^
Grain products and potatoes	17X	19X	Mind. 21
Vegetables and salad	7X	8X	Mind. 21
Fruits	5X	4X	Mind. 14
Milk and milk products	14X	8X	Mind. 14
Meat, sausages and fish	3/4/1X	5.5/6/0.9X	Max. 3X meat & sausages; min.2XFish
Oils	x	x	Colza oil
Giblets	2X	_	_
Cakes with oat bran	2X	_	_
Pulses	0.5X	0.3X	_

^a^DGE Qualitätsstandard in stationären Senioreneinrichtungen http://www.dge.de

## Discussion

With our study, we could indeed show that it is possible and feasible to improve the iron intake of geriatric patients with simple, easily available and accepted iron dense foods. The use of traditional and well-accepted food items embedded in the whole habitually consumed diet leads to a significantly improved iron intake. With our design, we were able to close the gap between the current and the recommended iron intake of geriatric patients. Furthermore, we succeeded in showing that a long-term food-based iron therapy is suited to improve the clinically relevant iron-deficiency anemia in geriatric patients. In the intervention group, the introduced way to increase the oral iron intake of geriatric patients was very practicable, particularly as the concept of the “meals forum” led to recipes well accepted by the patients. The acceptance of the chosen iron dense food items in the real life experience of the daily meals permitted to observe over the1-year period whether the intervention had the expected effect. As anticipated, it became apparent that the body’s iron availability improved. With the help of the significantly improved transferrin concentration, we concluded that in anemic patients the iron metabolic rate increased. Accordingly, the hemoglobin concentration increased and the anemia prevalence decreased. At the same time, it became apparent that in non-anemic patients, the body-stored iron increased too, and the ferritin concentration improved. Therefore, we drew the conclusion that the food-based intervention with iron dense foods in the daily meals in geriatric institutions showed the desired effects.

The feasibility of increasing iron rich foods may be also improved by the addition of additional meets and sausage, which may be an acceptable alternative to liver and legume rich foods. The menu plan habits of the additional consulted comparison center showed that improved diet and increased frequency of offering meets and sausages was associated with improved health and nutrition generally, including improved hemoglobin concentration. We assume that generally the offering of iron rich foods, plant or animal based, could led to the improved hemoglobin concentration. Our assumption rests on the fact that meat and sausages are rich in heme iron. As heme iron is absorbed well [8, 25] and intact [7, 10] the bioavailability of heme iron is good [26]. Moreover, it is well documented that the bioavailability of heme iron is remarkably good when iron storages are low [26]. Therefore, we conclude that patients with iron deficiency anemia benefit most from the offering of meat and sausage.

However, with our intervention we were able to achieve the same effect with a balanced, mixed diet. The menu plan was well adapted to the recommendations so that it can be assumed that this contributed to an overall well balanced nutrient intake [11, 22]. As regards the iron supply, the offered animal and plant based iron dense foods were in a balanced form, as recommended by nutritional societies [21]. Although it is well documented that non-heme iron is not well absorbed by the body, and its bioavailability varies greatly [7], we also could achieve an improved hemoglobin concentration. We consider that this was primarily because when iron levels are low, the bioavailability of non-heme iron improves [26]. Moreover, published data tells us that when the body iron levels are low, non heme iron is used in the same way as heme iron, since both sources feed into a common regulatory pathway triggered by a common signal in the intestines [10].

Furthermore, we assume that the study subjects’ overall health status improved over the year of observation. We assume that through the iron dense foods offering the vicious circle of a not sufficient iron intake and anemia prevalence can be broken. The bioavailability of iron in the offered foods was well enough for improving the hemoglobin concentration and therefore the health status of the geriatric patients. Therefore, based on the facts described above, iron dense food-offerings should be an integral part in the anemia management of institutionalized geriatric patients, particularly to improve their health status.

Nevertheless, this study does have some limitations. Mainly, the sample size was small. However, it was difficult to find geriatric patients that will be willing or able to give informed consent. Only 99 of 620 patients give their full written consent. Many of the patients have no interest to take part in a study like this one or have advanced dementia. Anyhow, as the hemoglobin concentration is the most meaningful parameter in assessing the prevalence of anemia [2] we think our conclusions are well founded.

However, the added value of this parallel, open, single group, pre-and post-oral nutrition intervention study is that to our knowledge this is the first study of this kind to investigate the associations between dietary iron intake through iron-dense foods and anemia in a sample population of geriatric patients. We clearly demonstrated that the iron intake through iron dense foods has its impact on the iron metabolism and the anemia prevalence in elderly patients. An additional strength of our study design is that we analyzed dietary patterns, which considered the entire diet and combinations of ingested foods. This approach may enhance our understanding of how a given diet affects iron deficiency.

## Conclusion

Geriatric patients with anemia or low iron level respectively benefit mostly from a diet rich in iron-dense-food. Enhanced access to such food can correct iron deficiency anemia. In geriatric institutions iron deficiency anemia must receive more attention when foods low in iron are served. The offered foods should be iron dense enough to avoid the onset of iron deficiency anemia.

## Abbreviations

BLS: Bundes-Lebensmittel-Schlüssel
D-A-CH: Deutsch-Austria-Schweiz
DGE: Deutsche Gesellschaft für Ernährung
MMSE: Mini-mental-status-test
WHO: World health organisation
